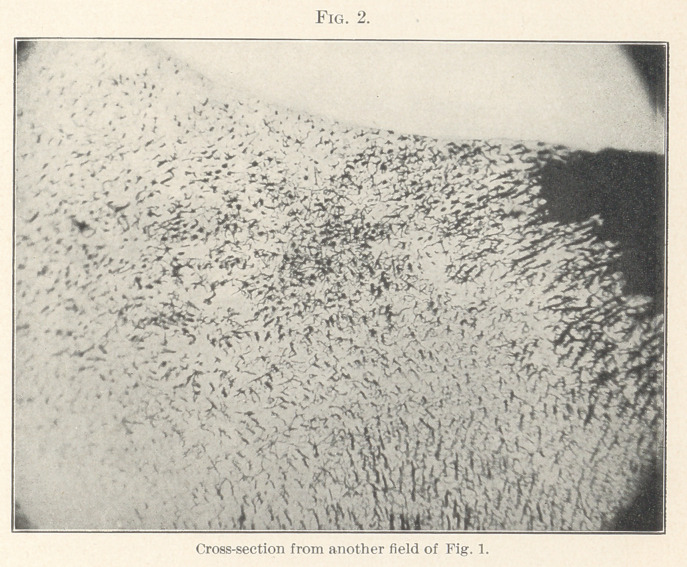# The Reticulum in Dentine

**Published:** 1902-12

**Authors:** John S. Engs

**Affiliations:** Oakland, Cal.


### THE RETICULUM IN DENTINE.

BY JOHN S. ENGS, D.D.S., OAKLAND, CAL.

On page 319 of the International Dental Journal for 1895, we find an article by F. A. Roy, entitled "A Reply to a Review of Bödecker's Book." The writer of that article took exceptions to the reviewer's conclusions, and prophesied that some day photographs would be made showing the reticulum of Heitzmann as Heitzmann describes it. One of the objections taken to the evidence presented in the book was, that the illustrations of the reticulum were all from drawings made by Heitzmann himself, and that drawings, however well executed, cannot carry conviction.

Between pages 632 and 633 of the International Dental Journal for 1892 will be found two plates made from drawings by Heitzmann. The article which they accompany ends with these words: "My intention in exhibiting these two specimens is to stop short all further doubts as to the reticular structure of the basis-substance of dentine. In face of the facts presented, I am entitled to expect such a result."

While these illustrations show a net-work of processes from the main tubules, they certainly do not present any such appearance as is shown in other illustrations of the reticulum made by him, taking, for instance, Fig. 6, on page 700 of the JOURNAL for the same year. This shows a uniform reticulum in the cementum, and is perhaps a better illustration of his claims than are the two plates previously referred to.

If the only objection to the reticulum as described by Heitzmann lay in the fact that enthusiasm and a skilful draughtsman may prompt the eye to see more than really exists, then the evidence furnished by the camera in recording what the microscope reveals should be sufficient to satisfy such objection, but he claims more than I think it possible for any camera to show.

It seems rather unfortunate that much of the work in dental histology has been upon decalcified or semidecalcified specimens. We could hardly expect a staining reagent to act the same upon a substance of glue-like consistence as it would upon the same substance when thoroughly impregnated with calcareous matter. In my work upon dental tissues I have endeavored so far as possible to preserve the natural relation and condition of the parts. This necessitated the use of investment methods such as would hold the tissues in place during the process of grinding or cutting in the microtome. In no case did I decalcify my specimens.

That my results might be uniform upon embryonic as well as upon fully developed and adult teeth, I observed the same technique in staining. This consisted of a combination of Golgi's with a carmine stain.

To obtain sections of embryonic teeth the microtome was used, the specimens being first embedded in paraffin. Owing to the advanced state of calcification the sections were somewhat broken, but in places the enamel attached to the dentine remains unbroken, being merely separated from the dental pulp.

The sections of embryonic teeth show the tubules of the dentine stained black, the silver salt probably giving what is known as a primary stain, or precipitate in the tissue.

If the specimen is overstained, as we find in sections made from surface cuttings, a diffusion of the stain seems to have taken place, resulting in obliteration of detail.

This is often seen near the border of the pulp and in the outer layer of the cementum in formed as well as in forming teeth. If the section is made sufficiently thin, however, the tubules will be clearly seen, well defined from the matrix, which remains practically unstained. Those processes which pass outward from the dentine into the enamel, cutting diagonally across the enamel-rods, are also stained black. The formed enamel, unlike the basis-substance of the dentine, is stained a deep pink by the carmine.

If the "so-called reticulum," which Dr. Andrews believed to be really the "substructure of connective-tissue fibres," existed as such, to afterwards become calcified, we would naturally look for some trace of it in the early stages of development in the embryo. I do not find it, but I do find, as previously stated, the dentinal tubules with their dichotomous endings and lateral branches, apparently of no greater dimension than in specimens taken from fully developed teeth. The lateral branches in some instances are seen to have a number of fine subdivisions which present a moss-like appearance, but I do not find a uniform net-work throughout the substance of the dentine.

The dentine of deciduous teeth presents the same appearance as that of permanent teeth, as regards the tubules and their branches.

As ordinarily seen, the dentinal tubules present the appearance of unbroken lines, passing outward from the pulp-chamber to the border of the enamel and cementum; here they divide into two or more branches, which in some instances appear to enter the interglobular layer. Long branches from the main tubule are in some instances given off; these run parallel to it, and may appear at any place between the border of the pulp and the interglobular layer.

Specimens stained with silver salts show other, shorter branches, which pass outward from the main tubule into the basis-substance of the dentine. These do not run parallel to the main tubule, and are apparently lost in the substance of the dentine. Not in all silver-stained specimens do they appear, but I attribute this to the failure of the stain to act, rather than to their non-existence.

Several specimens which I recently obtained from permanent and deciduous teeth show very beautifully, and demonstrate more clearly than do any illustrations which I have so far seen these lateral branches, which begin at the border of the pulp-chamber and crop out at intervals all the way to the intergranular layer.

In addition I find these branches to be *subdivided.* This is shown in the illustrations which accompany this article. Fig. 1

Fig. 1.

Longitudinal view near pulp-chamber.

Fig. 2.

Cross-section from another field of Fig. 1.

is a longitudinal view taken near the pulp-chamber. The specimen was ground from the palatal root of a sixth-year molar extracted from the mouth of a boy nine years of age. The field is midway of the root. Fig. 2 is a cross-section view taken from another field in the same specimen. Here we see a most intimate interlacing of the lateral branches.

Dr. Andrews says that the semicalcified tissue next the pulp is more readily acted upon by silver stains than is the fully calcified portion of the basis-substance. Looking at these photographs casually, this would appear to be so, but in places where the specimen is ground thinner the ends of the tubules are clearly defined from the basis-substance, showing that it has not primarily taken the same stain as the tubules.

I do not present this article to champion the views of Dr. Heitzmann or his school, but to show that I have obtained certain results in my work, photographs of which demonstrate a more intricate net-work of branches from the main tubules in the dentine than is generally believed to exist. These branches I believe to contain the same substance as the main tubules, that is, protoplasm.

In addition, let me state that I have observed in some of my specimens a certain tinting of the basis-substance of the dentine. I find this in that portion of the basis-substance farthest removed from the pulp-chamber, what we would consider fully calcified matrix. Being nearer the surface it was longer exposed to the action of the stain. Would this not suggest channels of communication other than those which I have demonstrated?

## Figures and Tables

**Fig. 1. f1:**
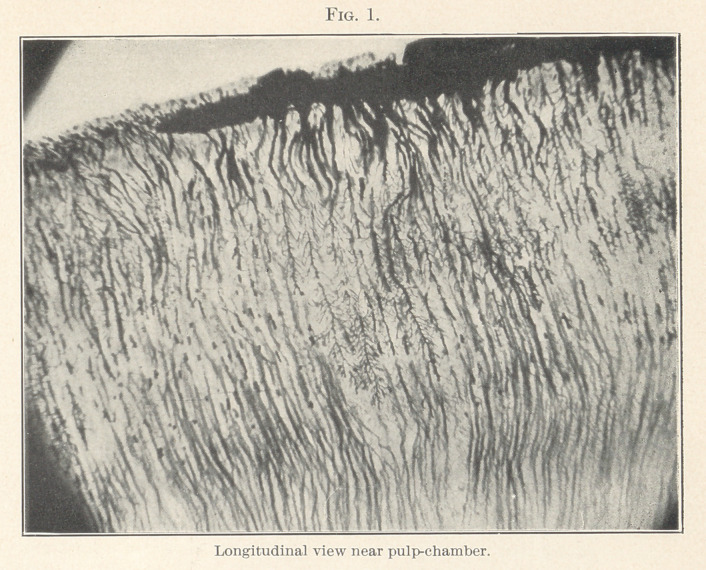


**Fig. 2. f2:**